# Sanger and Next Generation Sequencing Approaches to Evaluate HIV-1 Virus in Blood Compartments

**DOI:** 10.3390/ijerph15081697

**Published:** 2018-08-09

**Authors:** Andrea Arias, Pablo López, Raphael Sánchez, Yasuhiro Yamamura, Vanessa Rivera-Amill

**Affiliations:** AIDS Research Infrastructure Program, Ponce Health Sciences University-Ponce Research Institute, Puerto Rico 00716-2348, USA; aarias@psm.edu (A.A.); plopez@psm.edu (P.L.); rsanchez@psm.edu (R.S.); bonyamam@gmail.com (Y.Y.)

**Keywords:** HIV-1, blood compartment, plasma, PBMCs, memory CD4^+^ T-cells, Sanger Sequencing, Next-Generation Sequencing

## Abstract

The implementation of antiretroviral treatment combined with the monitoring of drug resistance mutations improves the quality of life of HIV-1 positive patients. The drug resistance mutation patterns and viral genotypes are currently analyzed by DNA sequencing of the virus in the plasma of patients. However, the virus compartmentalizes, and different T cell subsets may harbor distinct viral subsets. In this study, we compared the patterns of HIV distribution in cell-free (blood plasma) and cell-associated viruses (peripheral blood mononuclear cells, PBMCs) derived from ART-treated patients by using Sanger sequencing- and Next-Generation sequencing-based HIV assay. CD4^+^CD45RA^−^RO^+^ memory T-cells were isolated from PBMCs using a BD FACSAria instrument. HIV *pol* (protease and reverse transcriptase) was RT-PCR or PCR amplified from the plasma and the T-cell subset, respectively. Sequences were obtained using Sanger sequencing and Next-Generation Sequencing (NGS). Sanger sequences were aligned and edited using RECall software (beta v3.03). The Stanford HIV database was used to evaluate drug resistance mutations. Illumina MiSeq platform and HyDRA Web were used to generate and analyze NGS data, respectively. Our results show a high correlation between Sanger sequencing and NGS results. However, some major and minor drug resistance mutations were only observed by NGS, albeit at different frequencies. Analysis of low-frequency drugs resistance mutations and virus distribution in the blood compartments may provide information to allow a more sustainable response to therapy and better disease management.

## 1. Introduction

Differences in drug penetration in various compartments within HIV infected patients under antiretroviral therapy (ART) may account for viral compartmentalization, low-level viral replication, and mutation [1,2,3]. While ART therapy can suppress plasma HIV viral load below the detection limits, specific T cell compartments can remain as virus reservoirs, and the virus in different compartments have been shown to evolve independently [4,5,6]. The CD4^+^ T-cells are the primary targets of HIV, and due to the differences in drug distribution throughout the body, drugs resistance mutations may emerge, suggesting that HIV mutation patterns in plasma may not necessarily reflect those detected in the cell-associated compartment (CA) [7,8,9]. The factors discussed above together with patient non-adherence to treatment, drug toxicity, and antiretroviral treatment fatigue are associated with viral rebound [10]. Genotyping resistance testing has become an essential tool for improvement of HIV-1 ART outcomes [11]. HIV-1 genotyping is routinely performed by sequencing of the virus in the plasma of patients. Standard population-based Sanger sequencing may fail the detect low-frequency drug resistance mutations [12,13,14]. Next-Generation sequencing-based HIV assay is a new platform that allows monitoring low abundance viral variants, which may be involved in reduced susceptibility to ART treatment [15,16]. The objective of the present study was to evaluate the concordance of drug resistance mutations between plasma, peripheral blood mononuclear cells (PBMCs) and CD4^+^ memory T-cells (CD3^+^ CD4^+^ CD45RA^−^RO^+^) by using the Sanger sequencing and NGS platforms.

## 2. Methods

The current study was conducted in accordance with the Declaration of Helsinki, and the protocol was certified by the Institutional Review Board of the Ponce Research Institute to be exempt from the federal policy for the protection of human subjects under the provision of use of existing data and specimens (plasma and PBMC’s) (Protocol number: 140418-YY; date of approval: 03/28/2018). Peripheral blood samples (4 mL) were processed for viral load and complete blood counts from four HIV-1 positive individuals. The samples included in this study were selected from a bank of samples based on viral loads of at least 1000 copies/mL and availability of stored PBMCs and a minimum CD4^+^ T cell count of 350 cells/μL. Viral load was determined using the COBAS^®^ AmpliPrep/COBAS^®^ TaqMan^®^ HIV-1 Test version 2 using the COBAS^®^ AmpliPrep Instrument for automated specimen processing and the COBAS^®^ TaqMan^®^ 48 Analyzer for automated amplification and detection (Roche Diagnostics, Indianapolis, IN, USA). 

### 2.1. Cell Sorting

**Figure 1:**
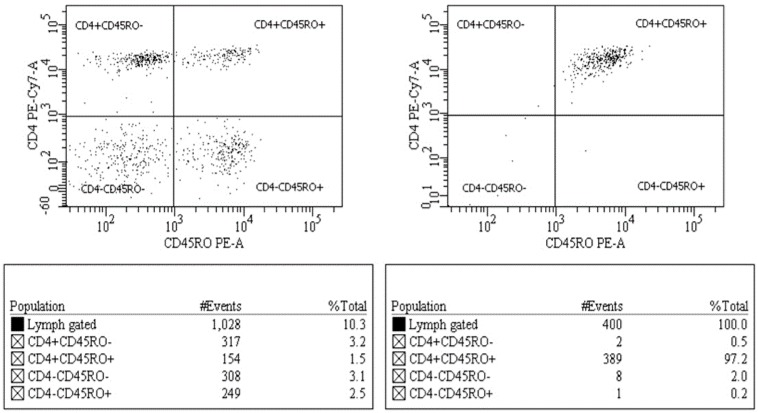
Analysis and cell sorting of CD4^+^ T-cells. The lymphocyte population was further identified as CD3^+^ CD4^+^ T-cells, each of which was then sub-classified into the memory (CD4^+^CD45RA^−^RO^+^) or naïve (CD4^+^CD45RA^+^RO^−^) subsets for cell-sorting. Purity of a sorted cell population was >95%.

The monoclonal antibodies used for identification of the cell markers were as follows: CD45RA-FITC, CD3-PerCP, CD4-PECy7, and CD45RO-PE. Respective isotype controls were used for identifying marker-positive populations. Analysis and cell sorting were performed by using a BD FACSAria (BD Biosciences, San Jose, CA, USA). The purity of CD4^+^ CD45RA^−^RO^+^ T-cells prepared by cell-sorting was consistent at >95% (Figure 1).

### 2.2. Nucleotide Acid Purification and PCR Amplification

After the cell sorting procedure, HIV-1 RNA and DNA was purified from the plasma and corresponding T-cell subsets, using QIAamp viral RNA Mini Kit and QIAamp DNA Blood Mini Kit (QIAGEN, Hilden, Germany), respectively, according to the manufacturer’s recommendations. Purified RNA and DNA were RT-PCR and PCR amplified by using the QIAGEN OneStep RT-PCR Kit (QIAGEN) and FastStart PCR Master (Sigma-Aldrich, Mannheim, Germany), respectively, according to our WHO accredited HIV-1 genotyping protocols. Briefly, first round RT-PCR conditions were as follows: 10 µL of purified RNA was reverse transcribed at 50 °C for 40 min, inactivation at 94 °C for 15 min, followed by 35 cycles of 94 °C for 30 s, 53 °C for 30 s, and 72 °C for 2 min with a final extension at 72 °C for 10 min. The first-round amplicon (2 µL) was re-amplified using the FastStart PCR Master (Sigma-Aldrich) under the conditions as follow: 95 °C for 15 min followed by 35 cycles of (94 °C for 15 s, 53 °C for 30 s, and 72 °C for 2 min) and a final elongation at 72 °C for 10 min. For DNA samples, both first round (10 µL of purified DNA) and second round (2 µL of first round PCR product) PCR amplification was carried out using the FastStart PCR master as described above.

Table 1 lists the primers used for the PCR (Sanger/NGS) procedures. The conditions of PCR to NGS procedures were similar to Sanger with some modifications in the amplification step (94 °C for 30 s, 55 °C for 30 s, and 72 °C for 2 min). Sanger sequences were determined by using an ABI 3730xl automated DNA Analyzer sequencer (Thermo Fisher Scientific Waltham, MA, USA). 

### 2.3. Sequencing and Phylogenetic Analyses

**Figure 2:**
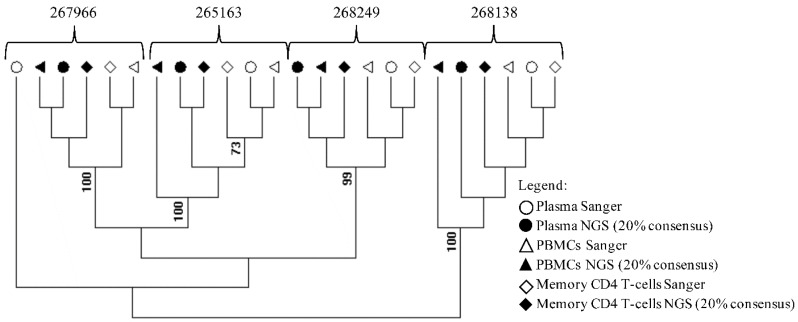
Phylogenetic assessment of cross-contamination in study samples. An HIV-1 *pol* gene (protease and reverse transcriptase) maximum likelihood tree was created by using MEGA software v6 from samples processed by Sanger- and NGS-based methods (consensus 20%). The blood compartment and sequence methodology are indicated by symbols.

The RECall software (beta v3.03, The University of British Columbia, Vancouver, BC, Canada) was used to align and edit the sequences [17]. Drug resistance mutations were evaluated by using the Stanford HIV database [18]. To evaluate possible sample contamination maximum likelihood phylogenetic tree of HIV-1 *pol* gene (protease and reverse transcriptase) from samples processed by Sanger sequencing and NGS-based methods (consensus 20%) was created by using MEGA software v6 under a General Time-Reversible (GTR) nucleotide substitution model with a gamma-distributed rate variation, which was suggested by Modeltest, and a re-sampling process (100 bootstraps) (Figure 2) [19]. The Sanger sequences and NGS sequences at the 20% threshold were submitted to Gen Bank with accession numbers: MH328213-MH328229, MH496618-MH49624. The NGS sequences were deposited at the National Center for Biotechnology Information Sequence Read Archive with accession number: PRJNA479460.

For NGS library construction, the first round PCR was re-amplified using amplicon primers containing the MiSeq (Illumina, Inc., San Diego, CA, USA) adapter sequences and the HIV-1 protease/reverse transcriptase (RT) locus-specific sequences. The samples were processed according to the 16S Metagenomic Sequencing Library Preparation guide (Illumina, LLC, Inc., San Diego, CA, USA) [20]. Briefly, the second round PCR was cleaned using magnetic beads and 80% ethanol, a third round PCR was performed to add unique barcodes to each sample, and the PCR product was cleaned one more time with magnetic beads and 80% ethanol. The barcoded amplicons were quantified using a Qubit 2.0 fluorometer and a Qubit dsDNA HS assay kit (Thermo Fisher Scientific Waltham, MA, USA)) and normalized to a 4 nM concentration. After normalization, samples were pooled, and diluted to 4 pM and heated to 96 °C for 2 min and 30 µL of the library were substituted with PhiX (12.5 pM). The denatured library was loaded on the Illumina MiSeq chip and a sequencing run of 2 × 250 bp MiSeq paired-end was performed. The resulting fastq files, Read 1 and Read 2 from each sample, were merged using FLASH software with the -M 550 and -O parameters [21]. The merged files were quality filtered using Trim Galore! with a minimal length of 360 bp, the option for filtering the Illumina adapter sequences (--illumina) and minimal error rate of 0.05 [22]. The InDelFixer aligner was used for aligning the reads to the reference sequences with the following options: -q 30, -illumina and -sensitive options [23]. Each sample has three fragments, protease (codon 1 of protease to 21 of RT), RT beginning (codon 15 to 141 of RT) and RT middle (codon 125 to 267 of RT) and all fragments were aligned to their corresponding sequences of the pol gene using the HIV-1 HXB2 reference sequence. The resulting sam files were converted to bam files, sorted and indexed using samtools, and the sorted bam files were converted to fastq files using the bamtools software [24,25]. The resulting fastq files were analyzed with Hydra Web (Government of Canada, Ottawa, Canada) with 20% and 5% sensitivity threshold for reporting low-frequency variants [26,27,28] We used the default target coverage (10,000 reads) and filtering settings (length cutoff: 100; score cutoff: 30) in HyDRA [29].

## 3. Results and Discussion

Sorting of CD4^+^CD45RA^−^RO^+^ T cells resulted in greater than 95% purity, indicating the isolation of the desired T cell population (Figure 1). Phylogenetic analysis of sequences generated using Sanger and NGS at the 20% threshold revealed that all samples formed well-defined clusters (Figure 2). Only the Sanger plasma sample from 267966 separates form its cluster.

Table 2 summarizes the plasma HIV-1 viral loads, CD4^+^ T-cell counts and PCR amplification results of the four samples included in this study. Overall, our results show a high correlation between Sanger sequencing and NGS results between intra-patient cell-free (CF) and cell-associated (CA) virus by using NGS at 20% consensus. However, in sample 267966 major resistance mutations (M184V, E138EA) were only detected in CA virus (NGS/Sanger) and plasma NGS (Table 2). 

The mutations were detected consistently in CA virus when comparing both methodologies. The discrepancy between CF and CA may result from the low viral load in the sample 267966 as demonstrated by others [30,31,32]. The nucleoside reverse transcriptase inhibitor (NRTI) mutation M184V causes high resistance to lamivudine (3TC) and emtricitabine (FTC), meanwhile, the non-nucleoside reverse transcriptase inhibitor mutation E138EA causes low-level of resistance to rilpivirine (RPV) [33,34,35]. Neither Sanger sequencing nor NGS at the 20% consensus identified the major resistance mutations in CF and CA virus in the samples of 268138, 275163 and 268249 (Table 2). Some accessory mutations, not currently included in the list of surveillance drug resistance mutations (SDRMs) were only observed by NGS, albeit at a different frequency in the data analysis by using 5% threshold (Table 3). In the sample 267966, the mutations M184V, E138A and E138EA were observed at high frequency by NGS in both CF and CA virus. Interestingly, the V179I mutation was only observed at high frequency (41.47%) in NGS-PBMCs. The accessory mutation A71V, associated with increased viral replication, was detected by Sanger and NGS in all compartments of sample 268138. The highly polymorphic accessory mutation K20R, associated with increased replication fitness in strain with PI-resistance mutations, was observed in all compartments of the sample 275163 by NGS only when using the 5% consensus [36]. Also, Sanger did not detect the accessory mutation L10V in the sample 268249, while it was detected at low-frequency (12.69%) by NGS (Table 3).

The purpose of this study was to assess the concordance between Sanger sequencing and NGS in the identification of drug resistance mutations in other compartments in addition to plasma. Overall, our results show a high correlation between Sanger sequencing and NGS results between CF and CA at the 20% threshold. We only detected one exception in the plasma from sample 267966, which is the sample with low viral load (1450 copies/mL) [37]. As expected, we observed additional drug resistance mutations in the 5% threshold analysis, and there was also a correlation between the results obtained by Sanger sequencing and NGS. While virus recovered from plasma is most commonly used for HIV-1 Sanger genotyping, HIV-1 in T-cell compartments of the same patient often present different patterns of mutations and high rates of recombination [38,39,40,41]. Previous studies demonstrated that memory CD4^+^ T-cell compartment in HIV-1 infected individuals under ART treatment contain high levels of proviral HIV-1, which makes the most significant contribution to the viral reservoir [42,43,44]. The prevalence of high frequency of mutations only in CA virus may be interpreted as the consequence of cumulative ART history of each patient [45,46]. Even though previous studies have indicated minor differences in drug resistance mutation patterns between HIV-1 in plasma versus viral cDNA in PBMCs of the same individual, it is assumed that both sources generally yield identical genotyping results [32,47,48]. Nevertheless, low-frequency mutations may emerge in patients with inadequate treatment management [49,50,51]. Recently, NGS sequencing has been proposed as an alternative to sequencing-based methods to identify mutations associated with resistance to antiretrovirals [52,53]. The NGS-based HIV assay is a new platform that allows the monitoring of low abundance viral variants. While the significance of these variants is presently unknown, they may be involved in reduced susceptibility to ART treatment. This technology reduces the turnaround time, and cost per nucleotide sequenced dramatically [15,16,54]. Analysis of low-frequency drug resistance mutations and virus distribution in the blood compartments may provide information allowing a more sustainable response to therapy and better disease management. The observation that in some cases HIV-1 in different compartments may have different combinations of mutations indicate that pro-viral DNA in PBMCs and CD4^+^CD45RA^−^RO^+^ memory T-cells may affect the genotyping results by contributing with additional drugs resistance mutations. Though recent studies raised questions regarding the clinical utility of NGS for genotyping purposes [55,56], the analysis of low-frequency drug resistance mutations and virus distribution in the blood compartments may provide information allowing a more sustainable response to therapy and better disease management. In newly diagnosed patients with no drug experience, analyzing the blood compartments using NGS may provide information on transmitted drug resistance mutations. Additional research is needed to determine the significance of the low abundance mutations detected by NGS.

## 4. Conclusion

We compared HIV drug resistance mutation genotyping by Sanger and NGS methodologies in plasma and other blood compartments. Our results show a high correlation between the two methodologies for detecting major DRMs in the various compartments from the same patient at the 20% threshold. At the 5% threshold, we also detected a correlation in the detection of both surveillance and non-surveillance mutations. Also, a low-frequency drug-resistance mutation was detected only by NGS in the cell-associated compartment. The clinical relevance of the detection of cell-associated low-frequency drug resistance mutations by NGS needs to be assessed.

## Figures and Tables

**Table 1:** Primers used for RT-PCR and PCR procedures.

Sanger
First Round Forward (Protease):5′-TGAARGAITGYACTGARAGRCAGGCTAAT-3′ Reverse (Protease): 5′-AYCTIATYCCTGGTGTYTCATTRTT-3′ Forward (RT): 5′-TTTYAGRGARCTYAATAARAGAACTCA-3′ Reverse (RT): 5′-CCTCITTYTTGCATAYTTYCCTGTT-3′
Second Round Forward (Protease): 5′-YTCAGRCAGRCCRGARCCAACAGC-3′ Reverse (Protease): 5′-CTGGTGTYTCATTRTTKRTACTAGGT-3′ Forward (RT): 5′- TTYTGGGARGTYCARYTAGGRATACC-3′ Reverse (RT): 5′- GGYTCTTGRTAAATTTGRTATGTCCA-3′
NGS
PR-INNER_F 5′-TCGTCGGCAGCGTCAGATGTGTATAAGAGACAGCTTTAACTTCCCTCAGGTCACTCT-3′ RT-1_R 5′-GTCTCGTGGGCTCGGAGATGTGTATAAGAGACAGGTCAATGGCCATTGTTTAACTTTTGG-3′ RT-1_F 5′-TCGTCGGCAGCGTCAGATGTGTATAAGAGACAGCCAAAAGTTAAACAATGGCCATTGAC-3′ PRNEWIN_R 5′-GTCTCGTGGGCTCGGAGATGTGTATAAGAGACAGCTGGTGTYTCATTRTTKRTACTAGGT-3′ 5FP127_F 5′-TCGTCGGCAGCGTCAGATGTGTATAAGAGACAGATACTGCATTTACCATACCTAG-3′ 3F262_R 5′-GTCTCGTGGGCTCGGAGATGTGTATAAGAGACAGTCCCACTAACTTCTGTATGTC-3′

**Table 2:** Summary of sample viral load, CD4^+^ T-cell counts and the mutations detected by Sanger sequencing and Next-Generation Sequencing. High correlation between Sanger sequencing and NGS-based HIV assay (consensus 20%) was observed in major resistance mutations analyses.

Samples	Protease Major Resistance Mutations	RT Major Resistance Mutations
267966 (Female) Viral load: 1450 copies/mL CD4^+^ T cell counts: 649 cells/µL Antiretroviral therapy: Yes		
Plasma Sanger	none	none
Plasma NGS (consensus 20%)	none	M184V, E138EA
PBMCs Sanger	none	M184V, E138A
PBMC’s NGS (consensus 20%)	none	M184MV, E138EA
CD4^+^ memory T-cells Sanger	none	M184V, E138A
CD4^+^ memory T-cells NGS (consensus 20%)	none	M184V, E138A
268138 (Male) Viral load: 12,500 copies/mL CD4^+^ T cell counts: 937 cells/µL Antiretroviral therapy: Not available		
Plasma Sanger	none	none
Plasma NGS (consensus 20%)	none	none
PBMCs Sanger	none	none
PBMCs NGS (consensus 20%)	none	none
CD4^+^ memory T-cells Sanger	none	none
CD4^+^ memory T-cells NGS (consensus 20%)	none	none
275163 (Female) Viral load: 16,105 copies/mL CD4^+^ T cell counts: 369 cells/µL Antiretroviral therapy: Yes		
Plasma Sanger	none	none
Plasma NGS (consensus 20%)	none	none
PBMCs Sanger	none	none
PBMCs NGS (consensus 20%)	none	none
CD4^+^ memory T-cells Sanger	none	none
CD4^+^ memory T-cells NGS (consensus 20%)	none	none
268249 (Female) Viral load: 32,468 copies/mL CD4^+^ T cell counts: 511 cells/µL Antiretroviral therapy: Yes		
Plasma Sanger	none	none
Plasma NGS (consensus 20%)	none	none
PBMCs Sanger	none	none
PBMCs NGS (consensus 20%)	none	none
CD4^+^ memory T-cells Sanger	none	none
CD4^+^ memory T-cells NGS (consensus 20%)	none	none

**Table 3:** Mutations detected by Next-Generation Sequencing. The threshold for data analysis was set at 5%. Protease and RT genes were analyzed and the table includes the gene and compartment with drug resistance mutations detected at the 5% threshold. NP indicates that high frequency mutations were not present in the samples analyzed. Letters in bold indicate drug resistance mutations that were also detected by Sanger sequencing. (-) indicates not applicable.

Sample	Gene	Classification	Surveillance	WT	Position	Mutation	Frequency	Coverage
267966								
Plasma	**RT**	**NRTI**	**Yes**	**M**	**184**	**V**	**99.4**	**46,490**
	**RT**	**NNRTI**	**No**	**E**	**138**	**A**	**99.29**	**55,090**
PBMCs	**RT**	**NRTI**	**Yes**	**M**	**184**	**V**	**42.23**	**69,897**
	**RT**	**NNRTI**	**No**	**E**	**138**	**A**	**42.29**	**69,891**
	RT	Other	No	V	179	I	41.47	69,896
CD4^+^ memory T-cells	**RT**	**NRTI**	**Yes**	**M**	**184**	**V**	**99.12**	**54,457**
	**RT**	**NNRTI**	**No**	**E**	**138**	**A**	**99.38**	**54,452**
268249								
Plasma	PR/RT	-	-	WT	-	NP	-	-
PBMCs	PR/RT	-	-	WT	-	NP	-	-
CD4^+^ memory T-cells	PR	Other	No	L	10	V	12.69	9382
268138								
Plasma	**PR**	**Other**	**No**	**A**	**71**	**V**	**99.17**	**20,609**
PBMCs	**PR**	**Other**	**No**	**A**	**71**	**V**	**98.64**	**66,474**
CD4^+^ memory T-cells	**PR**	**Other**	**No**	**A**	**71**	**V**	**98.49**	**79,020**
275163								
Plasma	**PR**	**Other**	**No**	**K**	**20**	**R**	**23.13**	**49,598**
PBMCs	**PR**	**Other**	**No**	**K**	**20**	**R**	**97.03**	**46,084**
CD4^+^ memory T-cells	**PR**	**Other**	**No**	**K**	**20**	**R**	**96.9**	**33,222**
